# A systematic review and meta-analysis to determine the contribution of mr imaging to the diagnosis of foetal brain abnormalities *In Utero*

**DOI:** 10.1007/s00330-016-4563-4

**Published:** 2016-09-21

**Authors:** Debbie Jarvis, Cara Mooney, Judith Cohen, Diana Papaioannou, Mike Bradburn, Anthea Sutton, Paul D. Griffiths

**Affiliations:** 1 Academic Unit of Radiology, University of Sheffield, Sheffield, UK; 2 School of Health and Related Research (ScHARR) University of Sheffield, Sheffield, UK

**Keywords:** Ultrasound, Prenatal diagnosis, Magnetic resonance imaging, Foetal, Brain malformations

## Abstract

**Objectives:**

This systematic review was undertaken to define the diagnostic performance of in utero MR (iuMR) imaging when attempting to confirm, exclude or provide additional information compared with the information provided by prenatal ultrasound scans (USS) when there is a suspicion of foetal brain abnormality.

**Methods:**

Electronic databases were searched as well as relevant journals and conference proceedings. Reference lists of applicable studies were also explored. Data extraction was conducted by two reviewers independently to identify relevant studies for inclusion in the review. Inclusion criteria were original research that reported the findings of prenatal USS and iuMR imaging and findings in terms of accuracy as judged by an outcome reference diagnosis for foetal brain abnormalities.

**Results:**

34 studies met the inclusion criteria which allowed diagnostic accuracy to be calculated in 959 cases, all of which had an outcome reference diagnosis determined by postnatal imaging, surgery or autopsy. iuMR imaging gave the correct diagnosis in 91 % which was an increase of 16 % above that achieved by USS alone.

**Conclusion:**

iuMR imaging makes a significant contribution to the diagnosis of foetal brain abnormalities, increasing the diagnostic accuracy achievable by USS alone.

***Key points*:**

*• Ultrasound is the primary modality for monitoring foetal brain development during pregnancy*

*• iuMRI used together with ultrasound is more accurate for detecting foetal brain abnormalities*

*• iuMR imaging is most helpful for detecting midline brain abnormalities*

*• The moderate heterogeneity of reviewed studies may compromise findings*

## Introduction

Abnormalities of the foetal brain occur in approximately 25 per 10,000 births in the UK [1] and can result from environmental, chromosomal, genetic or acquired causes. Accurate diagnosis of foetal brain abnormalities is necessary to guide management of the pregnancy and facilitate parental counselling.

Ultrasound scanning (USS) is the primary diagnostic imaging method for screening of the pregnancy and considered the reference standard for imaging the foetus brain. There are occasions when technical limitations hinder clear visualisation of the foetal anatomy [2, 3] which led to the exploration of other diagnostic tests to supplement USS.

Advances in MR technology have meant initial technical restrictions in imaging the foetus with in utero magnetic resonance (iuMR) imaging have been overcome, experience within radiology has increased and a growing body of literature confirms increasing use of iuMR in diagnosing foetal brain abnormalities [4–7]. Despite this, the true clinical value of iuMR has not been established. Previous limited statistical evidence was unable to demonstrate, in terms of diagnostic accuracy, any benefit [8].

To our knowledge, there have been only two other recently published systematic reviews in which Rossi and van Doorn aimed to clarify the additional benefit of MRI in the diagnostic pathway when used in addition to USS [9, 10]. Rossi reviewed 13 studies and van Doorrn selected 27 studies for review. Despite similar aims and inclusion criteria only seven studies were included in both reviews. This could, along with date differences for searches, be due to the differences in exclusion criteria. The criteria used by Rossi excluded studies without an outcome reference diagnosis (ORD), non-English publications and those where data were reported in graphs or percentages. Van Doorns review excluded studies with a sample size of less than 20 and studies where diagnoses were inadequately described. We felt a new systematic review was justified in order to update the existing, to attempt to limit the number of studies excluded and to identify any other studies which may have been erroneously excluded.

The aim of this study is to answer the following question: Is the diagnostic accuracy of iuMR superior, equivalent or inferior to USS? We aimed to assess diagnostic accuracy of iuMR following antenatal USS through:

(a) Measurement of diagnostic accuracy of antenatal USS alone (i.e. prior to iuMR) in relation to an ORD determined by postnatal imaging, surgery or post-mortem examination

(b) Measurement of diagnostic accuracy of iuMR (following antenatal USS) relative to an ORD

Secondary aims were to determine if counselling and/or management of the pregnancy changes as a result of iuMR imaging and to identify the foetal brain anomalies for which iuMR is most useful.

## Methods

### Protocol

The protocol was written in accordance with the Preferred Reporting Items for Systematic Reviews and Meta-Analyses (PRISMA) [11] and registered with the International Prospective Register of Systematic Reviews (PROSPERO, CRD42015010265).

### Eligibility criteria

All study designs were considered eligible apart from case reports, reviews or commentaries.

#### Participants

Pregnant women who had undergone, due to suspicion of a brain abnormality, prenatal ultrasound and subsequent prenatal iuMR of their foetus’ brain and any findings confirmed by an ORD.

#### Reference standard

Reference standards accepted to confirm the outcome diagnosis were postnatal imaging (transcranial US, MRI or CT) and surgery or, in cases of foetal demise or neonatal death, autopsy and post-mortem MR imaging.

#### Exclusions

Studies not reported in English and translation was unavailable. If an English abstract was available these were scrutinised for relevant information, but limited data meant adherence to the inclusion criteria could not be certain.

### Search methods

We identified all studies in which iuMR imaging was used to supplement USS for imaging foetal brain abnormalities in utero using a sensitive search strategy of the following electronic databases using MesH and free-text terms as detailed in Appendix 1, adapting the strategy for each database.

Databases searched were Medline (via OVID) (1966 to present), EMBASE (via OVID) (1980 to present), Cochrane Register of Diagnostic Test Accuracy Studies (accessed 18/03/2015 and 02/10/2015) and Web of Science (1900 to present). In addition, we searched relevant journals, conference proceedings and examined reference lists of relevant and included studies.

Electronic searches were conducted in March 2015 without date restriction and later updated to identify all relevant papers up to September 2015.

### Data collection

#### Selection of studies

Screening of citations was completed independently by two reviewers (DJ, CM). Any disagreements were resolved by consensus. Where only abstracts were available, attempts were made to contact authors for full reports. If the same data had been published in more than one publication, the most up to date or complete study was selected.

**Fig. 1:**
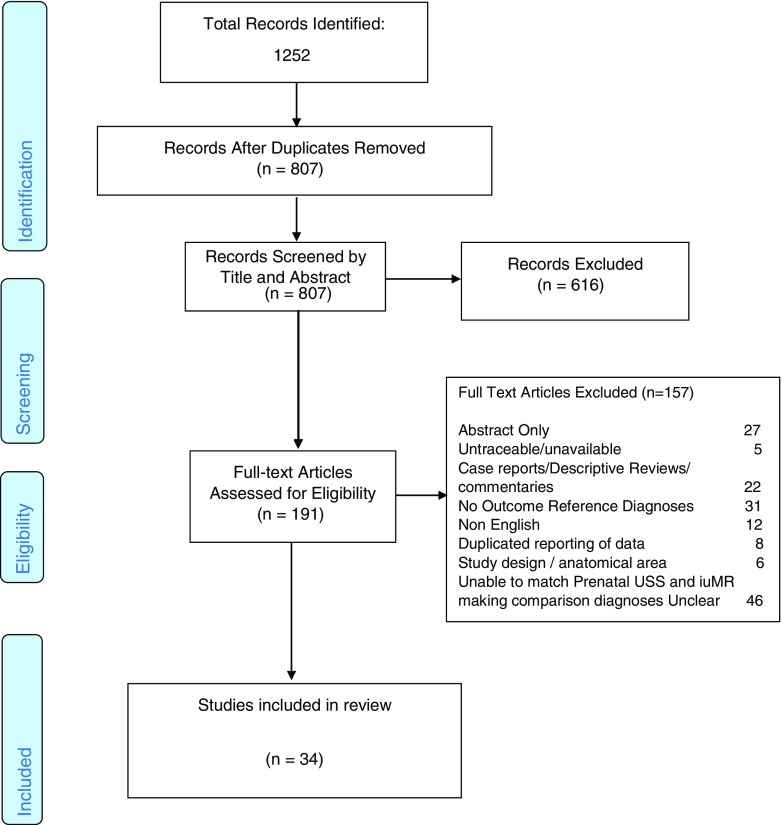
PRISMA flowchart of study selection and exclusions

A PRISMA flowchart was used to document and report any decisions made during the study selection process [9] (Fig. 1).

#### Assessment of methodological quality of included studies

Included studies were assessed independently for methodical quality (DJ and CM) using a modified Quality Assessment of Diagnostic Accuracy Studies (QUADAS 2) tool [12]. Studies were rated in terms of bias risk and applicability using signalling questions to score the four key domains—Patient selection, Index tests, Reference standard and Flow and Timing. Studies were scored as “Yes”, “No” or “Unclear” for each checklist item. Additional signalling questions were introduced for both study design and index tests. These were to determine prospective versus retrospective design and details regarding USS and iuMR technique and reporting as these were elements considered likely to introduce bias.

#### Data items and analysis

Study characteristics and outcomes were extracted independently (DJ and CM) and recorded using a data collection form (Appendix 2) which was piloted on three papers to ensure suitability. Characteristics noted for each study are listed in Appendix 2. The number of correct and incorrect diagnoses made by both USS and iuMR were also recorded as judged by the ORD confirmed by postnatal imaging, autopsy or surgery. Clinical examination was discounted as a reference standard as the majority of structural brain abnormalities are not apparent externally. Where studies reported the results of imaging from multiple anatomical areas, only results of the foetal brain were included.

It was anticipated that all studies would recruit only (or predominantly) foetuses with a brain abnormality diagnosed by USS, meaning the sensitivity and specificity of the imaging modalities could not be estimated because of the lack of foetuses without brain abnormality. Therefore, the analysis defined diagnostic accuracy for each modality as the percentage of cases where the diagnosis was confirmed by ORD. In foetuses with multiple abnormalities a primary diagnosis was identified as the abnormality with the most detrimental clinical outcome. In cases where both modalities identified the primary diagnosis but one provided a more specific diagnosis and/or additional information without fundamentally changing the primary diagnosis, our analysis assumed both modalities were correct but the nature of disagreements was subsequently investigated.

A meta-analysis of the diagnostic accuracy of iuMR in relation to USS was conducted using the Stata statistical analysis software [13]. For each study the odds ratio for the paired iuMR and USS accuracies and its standard error were computed using the method of Becker and Balagtas, using a 0.5 correction for zero cells [14, 15]. Odds ratios were combined using a random effects model and the *I* ^2^ statistic was used as an indicator of heterogeneity within the included studies [16, 17].

## Results

Our initial searches generated a total of 1252 potential studies with 807 remaining for additional scrutiny after duplicates were removed. Further screening resulted in 34 published studies for final inclusion [3, 18–50]. Categories for exclusion of full papers reviewed but rejected are listed in the PRISMA flowchart (Fig. 1).

### Study characteristics

The 34 studies, listed in Table 1, were published over a 20-year period (1994–2014). Nineteen were prospective [3, 18–35], 12 retrospective [36–47] and three unspecified [48–50]. All studies selected a consecutive cohort of patients with either a remit to investigate all foetal brain abnormalities (24 studies [3, 18–23, 29–32, 35, 39–41, 44–49]) or to investigate a more specific brain abnormality e.g. ventriculomegaly, corpus callosum anomalies (10 studies) [24–28, 34, 36, 38, 43, 50].

**Table 1:** Studies included in the review and their characteristics

Author, year	Title/objective	Country of study	Target population	Method of selection	Retrospective (R), prospective (P), not specified (NS)	Total number in study	Final number included in review
Amini et al. 2010	The clinical impact of fetal magnetic resonance imaging on management of CNS anomalies in the second trimester of pregnancy	Sweden	Foetuses with suspected CNS abnormality on USS	Consecutive	P	29	18
Benacerraf et al. 2007	What does magnetic resonance imaging add to the prenatal sonographic diagnosis of ventriculomegaly?	USA	Foetuses with VM on USS	Consecutive	P	26	13
Benoist et al. 2008	Cytomegalovirus-related fetal brain lesions: comparison between targeted ultrasound examination and magnetic resonance imaging	France	Foetuses with CMV infection	Consecutive	R	49	47
Blaicher et al. 2003	Magnetic resonance imaging in foetuses with bilateral moderate ventriculomegaly and suspected anomaly the corpus callosum on ultrasound scan	Austria	Foetuses with suspected VM and ACC on USS	Consecutive cases with VM and ACC	P	41	14
Colleoni et al. 2012	Prenatal diagnosis and outcome of fetal posterior fossa fluid collections	Italy	Foetuses with posterior fossa abnormality on USS	Consecutive fetuses with posterior fossa abnormalities	R	105	51
D'Ercole et al. 1998	Prenatal diagnosis of fetal corpus callosum agenesis by ultrasonography and magnetic resonance imaging	France	Foetuses with suspected ACC on US	Consecutive	P	14	8
Doneda et al. 2010	Early cerebral lesions in cytomegalovirus infection: prenatal MR imaging	Italy	Foetuses with CMV infection	Consecutive	P	38	13
Frates et al. 2004	Fetal anomalies: comparison of MR imaging and US for diagnosis	USA	Foetuses with abnormalities detected at US	Consecutive	P	27	16
Garcia-Flores et al. 2013	Fetal magnetic resonance imaging and neurosonography in congenital neurological anomalies: supplementary diagnostic and postnatal prognostic value	Spain	Foetuses with CNS abnormalities	Consecutive	R	28	24
Glenn et al. 2005	Fetal magnetic resonance imaging in the evaluation of fetuses referred for sonographically suspected abnormalities of the corpus callosum	USA	Foetuses with suspected CC abnormalities	Consecutive cases selected of foetuses with suspected CC abnormalities	R	10	r6
Hagmann et al. 2008	Foetal brain imaging: ultrasound or MRI. A comparison between magnetic resonance imaging and a dedicated multidisciplinary neurosonographic opinion	UK	Comparison of standard US, specialist US and MRI accuracy + change in management	Consecutive	R	51	12 (comparison of specialist US and MRI only)
Hamisa et al. 2013	Magnetic resonance imaging versus Ultrasound examination in detection of prenatal fetal brain anomalies	Egypt	Foetuses with suspected brain abnormality on USS	Consecutive	P	23	23
Hosny & Elghawabi 2010	Ultrafast MRI of the fetus: an increasingly important tool in prenatal diagnosis of congenital anomalies	Egypt	Foetuses with suspected brain abnormality on USS	Consecutive	NS	25	16
Ismail et al. 2002	Fetal magnetic resonance imaging in prenatal diagnosis of central nervous system abnormalities: 3-year experience	UK	Foetuses with suspected brain abnormality on USS	Consecutive	R	27	20
Kul et al. 2012	Contribution of MRI to ultrasound in the diagnosis of fetal anomalies	Turkey	Foetuses with suspected brain abnormality on USS	Consecutive	P	184	76
Malinger et al. 2004	Fetal brain imaging: a comparison between magnetic resonance imaging and dedicated neurosonography	Israel	Foetuses with suspected brain abnormality on USS	Consecutive	P	42	30
Malinger et al. 2011	Can syndromic macrocephaly be diagnosed in utero?	Israel	Foetuses with suspected macrocephaly on US	Consecutive	R	98	8
Manganaro et al*.* 2012	Role of foetal MRI in the evaluation of ischaemic-haemorrhagic lesions of the foetal brain	Italy	Foetuses with ischaemic-haemorrhagic lesions	Consecutive with inclusion criteria	P	271	13
Peruzzi et al. 2010	Magnetic resonance imaging versus ultrasonography for the in utero evaluation of central nervous system anomalies	USA	Foetuses with suspected CNS abnormality on USS	Consecutive	R	26	26
Phua et al. 2009	Magnetic resonance imaging of the fetal central nervous system in Singapore	Singapore	Foetuses with suspected CNS abnormality on USS	Foetuses who had an MRI	R	31	13
Resta et al. 1994	Magnetic resonance imaging in pregnancy: study of fetal cerebral malformations	Italy	Foetuses with suspected CNS abnormality on USS	Consecutive	P	15	11
Rubod et al. 2005	Role of fetal ultrasound and magnetic resonance imaging in the prenatal diagnosis of migration disorders	France	Foetuses with suspected migration abnormalities on US	Consecutive	NS	14	9
Saleem et al. 2009	Fetal MRI in the evaluation of fetuses referred for sonographically suspected neural tube defects (NTDs): Impact on diagnosis and management decision	Egypt	Foetuses with suspected NTD on US	Consecutive	P	19	19
Simon et al. 2000	Fast MR imaging of fetal CNS anomalies in utero	USA	Foetuses with suspected CNS abnormality on USS	Consecutive	P	73	23
Sohn et al. 2007	The usefulness of fetal MRI for prenatal diagnosis	Korea	Foetuses with suspected CNS abnormality on USS	Consecutive	R	30	6
Twickler et al. 2003	Second-opinion magnetic resonance imaging for suspected fetal central nervous system abnormalities	USA	Foetuses with suspected CNS abnormality on USS	Consecutive	P	72	72
Wang 2006	Fetal central nervous system anomalies: Comparison of magnetic resonance imaging and ultrasonography for diagnosis	China	Foetuses with suspected CNS abnormality on USS	Consecutive	NS	34	34
We et al. 2012	Usefulness of additional fetal magnetic resonance imaging in the prenatal diagnosis of congenital abnormalities	Korea	Foetuses with suspected brain abnormality on USS	Consecutive (8 years)	R	81	23
Whitby et al. 2004	Comparison of ultrasound and magnetic resonance imaging in 100 singleton pregnancies with suspected brain abnormalities	UK	Foetuses with suspected CNS abnormality on USS	Consecutive	P	101	100
Whitby et al. 2004	Corroboration of in utero MRI using post-mortem MRI and autopsy in foetuses with CNS abnormalities	UK	Foetuses with prenatal US and iuMRI and who underwent PM MRI	Consecutive	P	12	12
Yuh et al. 1994	MR of fetal central nervous system abnormalities	USA	Foetuses with suspected CNS abnormality on USS	Consecutive	P	22	19
Rajaswaran et al. 2009	Ultrasound versus MRI in the diagnosis of fetal head and trunk abnormalities	India	Foetuses with suspected head or trunk abnormality on USS	Consecutive fetuses with head or trunk abnormalities	P	40	30
Lipitz et al. 2010	Value of prenatal ultrasound and magnetic resonance imaging in assessment of congenital primary cytomegalovirus infection	Israel	Foetuses with CMV infection	Consecutive	P	38	35
Paladini et al. 2014	Accuracy of neurosonography and MRI in clinical management of fetuses referred with central nervous system abnormalities	Italy	Accuracy of US and MRI	Consecutive	R	834	126

USS was performed in a tertiary centre and/or conducted by foetal medicine experts in 21/34 studies [3, 18–21, 26–29, 31, 32, 35–38, 40–43, 47], in 12/34 it was either unclear or not specified [22–24, 30, 33, 34, 39, 45, 46, 48–50], and in one study [44] USS was performed in a routine clinical setting. Clear details regarding USS technique (transabdominal or transvaginal, views obtained) and equipment (manufacturer, transducer) were provided in 21 studies [18–20, 22, 24–26, 28, 32–37, 39, 40, 42, 43, 47, 48, 50]. The remaining 13 studies [3, 21, 23, 27, 29–31, 38, 41, 44–46, 49] provided minimal information or details were not given. Three out of 34 acknowledged technical difficulties in some cases which limited the USS [3, 28, 48]. The age range of foetuses reported across studies was 13–41 weeks gestation. Time delay between USS and iuMR was less than 2 weeks in 19/34 [3, 18–21, 23–25, 27, 30, 32, 33, 39, 41, 42, 44, 46, 47, 49] and not specified in 13/34 studies [22, 26, 28, 29, 31, 34, 36, 38, 40, 43, 45, 48, 50]. In two studies [35, 37] there were cases in which the time delay was greater than 2 weeks.

Experience of the clinician reporting the iuMR study was only available in 10/34 studies [20, 21, 25, 27, 28, 30, 32, 35, 37, 42], half of these quantified this in terms of years (between 1 and 15) the remaining gave a description of ‘experienced’. In two studies, the reporting radiologist was unaware of USS findings [21, 37]. Information regarding MR technique was reported in all papers including at least two of the following: manufacturer, sequences, types of receiver coils and patient positioning. Fast T2-weighted sequences were performed in all studies with some using additional sequences (e.g. T1, DWI, 3D and FLAIR). Early studies reported the use of fasting and sedation to achieve optimal imaging [22, 34].

### Methodological quality

**Fig. 2:**
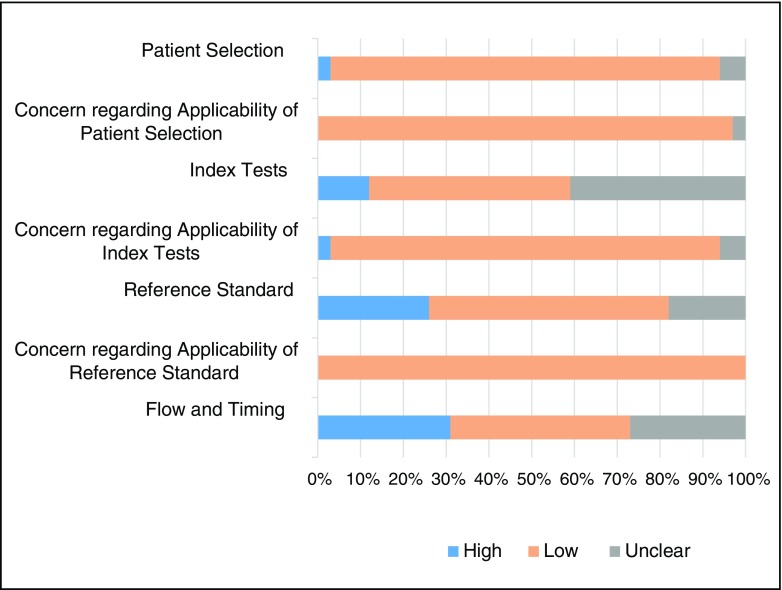
QUADAS risk of bias assessment

The methodological quality assessments using the Quadas 2 criteria are presented in Fig. 2. Risk of bias for patient selection and applicability was low in 31/34 (91 %) studies [3, 18–45, 47, 50], high in one (6 %) [46] and unclear in two [48, 49] with high risk of bias due to patient selection criteria not being defined and retrospective study designs. The risk of bias due to conduct and interpretation of the index tests was low risk in 15/34 (44 %) [3, 18, 20, 21, 25, 28, 30, 32, 35–37, 40, 42, 43, 47], high risk in 4/34 (12 %) [38, 44–46] and unclear in 15/34 (44 %) [19, 22–24, 26, 27, 29, 31, 33, 34, 39, 41, 48–50]. Assessment of potential bias introduced by the reference standard was considered low risk in 19/34 (56 %) studies [3, 18, 19, 21, 22, 24, 28–31, 35, 36, 38, 40, 44, 47–50], high risk in nine (26 %) [20, 27, 32–34, 41, 43, 45, 46] and unclear in 6/34 (18 %) [23, 25, 26, 37, 39, 42], as there were a proportion of cases within the study that did not have a confirmed outcome or it was determined by clinical examination. Bias in the flow and timing as judged by timing between USS and iuMR imaging or due to methods used for analysis of findings was deemed low in 15/34 (42 %) [3, 18, 19, 23–25, 30, 32, 33, 35, 39, 46, 47, 49], high in 11/34 (32 %) [21, 26, 31, 34, 36–42] and unclear in 9/34 (27 %) [20, 22, 27–29, 44, 48, 50].

### Diagnostic accuracy of US and MRI

The 34 included studies reported a combined total of 2530 foetuses (median 32.5, range 10–834) but of these 62 % (*n* = 1571) were excluded as they did not have an iuMR (*n* = 796), 542 did not have an ORD, were non-brain pathology (*n* = 159) or other exclusions (*n* = 74). Consequently this systematic review reports on the outcomes of 959 foetuses. In 6/34 studies [19, 28–30, 44, 49], all foetuses had an ORD, and combined contributed 186/959 to the analysis in this review (median 24.5, range 12–72). The remaining 773/959 (median 38, range 10–834) foetuses were from the outstanding 28 studies [18, 20–27, 30–43, 45–48, 50].

**Fig. 3:**
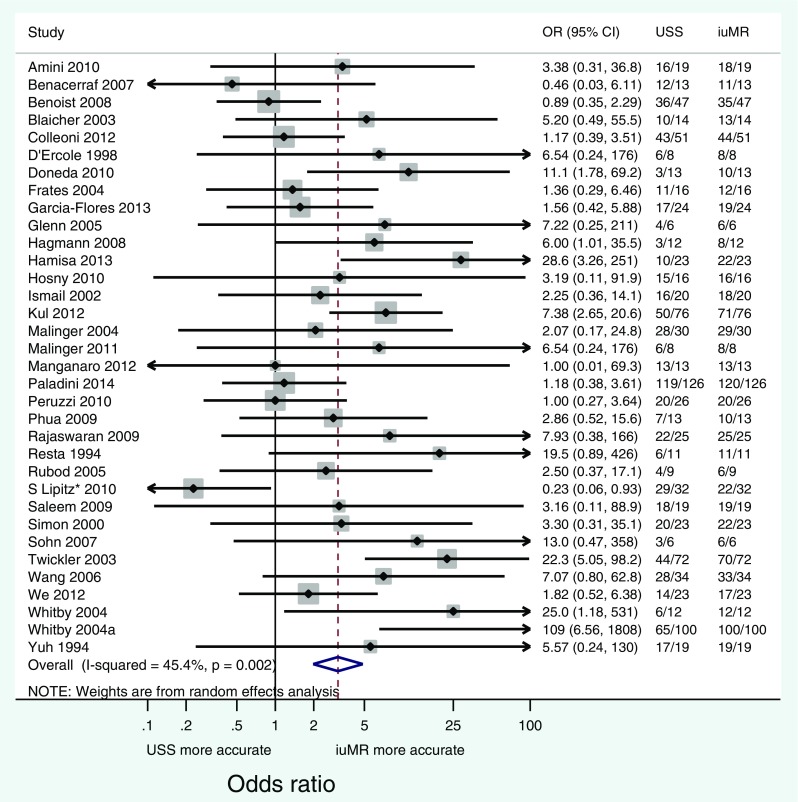
Forest plot showing the odds ratios of all studies (first author and date only) and overall odds ratio with confidence intervals

**Fig. 4:**
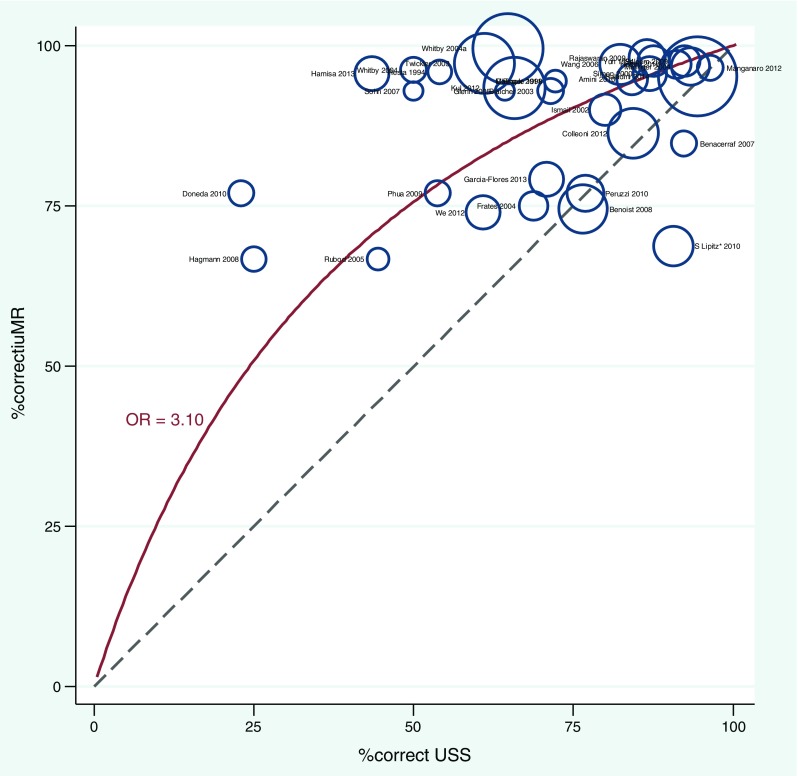
L’Abbe plot of diagnostic accuracy of USS and iuMR. Circle size is proportional to sample size of each study

The overall diagnostic accuracy combined across 34 studies was 75.2 % for USS and 91.0 % for iuMR (overall odds ratio = 3.10, 95 % CI 1.98 to 4.86, *p* < 0.0001; Fig. 3). Although individual studies were heterogeneous (*I* ^2^ = 45 %; *p* = 0.002), nearly all reported an improvement in diagnostic accuracy following iuMR. The data are also represented in the form of a L’Abbe plot (Fig. 4) in which the diagnostic accuracies of iuMR and USS are presented as percentages.

#### Agreement between USS and iuMR

The reports from USS and iuMR were in agreement and agreed with the ORD in 527/959 (55 %). USS and iuMR were in agreement but discordant with the ORD in 52/959 (5.5 %) foetuses (Table 1a and b, and 2).

**Table 2:** Results of the number and percentage of foetuses within each category of outcome

		Number	Percentage
1a	iuMR and USS agreed and correct	527	55
1b	iuMR and USS agreed but incorrect	52	5.5
2a	iuMR more exact/additional info to USS	146	15
2b	iuMR changed incorrect USS diagnosis	186	19
	Abnormalities identified correctly by iuMR but missed by USS	139	
	Abnormalities diagnosed by USS but correctly excluded by iuMR	47	
3a	USS more exact/additional info to iuMR	14	1.5
3b	iuMR incorrectly changed correct USS diagnosis	34	4
	Abnormalities diagnosed by USS but wrongly excluded by iuMR	10	
	Abnormalities overdiagnosed by iuMR that were absent on USS and ORD	24	
Total		959	

In 160/959 (16.5 %) foetuses iuMR and USS were in agreement regarding the primary diagnosis but additional information was added—either secondary diagnoses or a more concise/confident primary diagnosis given. In this category iuMR provided additional information in 146/959 (15 %) and USS provided additional information in 14/959 (1.5 %) cases as confirmed by ORD.

#### Disagreement between USS and iuMR

The diagnoses on iuMR and USS disagreed in 222 (23 %) cases. Of these, the iuMR was in agreement with the ORD in 186 (19 %), the majority of which were abnormalities undetected by USS (139/186, 75 %). The remaining 47/186 (25 %) were abnormalities reported by USS but correctly excluded by iuMR. In 34 cases the USS diagnosis was incorrectly overturned by iuMR, 10 of which were abnormalities wrongly excluded or missed by iuMR and 24/34 were abnormalities diagnosed by iuMR but not found by USS or on the ORD (Table 2b and 3b).

**Table 3:** Discordant diagnoses according to abnormality detected

Anomalies identified	Abnormalities identified correctly by MRI but missed by US	Abnormalities diagnosed by US but correctly excluded by MRI	Abnormalities diagnosed by US but wrongly excluded by MRI	Abnormalities overdiagnosed by MRI that were absent on US and ORD	US and MRI diagnoses both wrong (either missed or overdiagnosed)	All groups
Ventricular system (Ventriculomegaly, aqueduct stenosis)	5	10	1	1	6	23
Neural tube defects (Anencephaly, encephalocoele, myelomeningocele)	5	5	0	1	1	12
Cortical formation abnormalities (Hemi/megalencephaly, schizencephaly, lissencephaly, heterotopia, microcephaly)	21	3	3	5	14	46
Midline abnormalities (Holoprosencephaly, agenesis/hypogenesis of corpus callosum, absent cavum septum)	39	15	2	1	7	64
Posterior fossa (Abnormalities: mega cisterna magna, Blake’s pouch cyst, Dandy-Walker or variant cerebellar or vermian hypoplasia)	28	13	2	2	12	57
Vascular abnormalities (Haemorrhage, haematoma dural fistula aneurysm)	17	0	1	1		20
Destructive or mass cerebral lesions (Tumours, Cysts, PVL, other lesions, dysplasias)	24	1	1	13	11	50
Totals	139	47	10	24	52	

Table 3 presents the discordant diagnoses between USS and iuMR according to category of anomaly. The most frequent areas of disagreement were midline (24 %) and posterior fossa abnormalities (21 %). In particular agenesis of the corpus callosum and the Dandy Walker spectrum of abnormalities were frequently missed or, less frequently, wrongly identified on USS. The most frequently misdiagnosed anomalies on both USS and iuMR were cortical formation abnormalities (17 %) such as hemimegalencephaly, lissencephaly and heterotopia.

#### Changes in counselling and management

Eleven studies [3, 18, 28–31, 40, 41, 44, 47, 48] reporting on 186 foetuses specified the benefit of iuMR in terms of changes to counselling of parents or management of the pregnancy. These changes as a result of findings on iuMR affected 78/186 (41.9 %) foetuses.

## Discussion

This systematic review and meta-analysis demonstrates that using iuMR to support USS in the diagnosis of foetal brain abnormalities increases diagnostic accuracy by 16 % (75 % for USS alone and 91 % for iuMR as an adjunct). The heterogeneity of the included studies was moderate (*I* ^2^ = 45 %, *p* = 0.002) according to the definitions of Higgins et al. [51], suggesting methodological and clinical variability and inconsistency in the measurement of outcomes within each study. Although investigation of heterogeneity is recommended [51], the ability to do so is compromised by the lack of reporting (and indeed quantification) of all the ways in which studies differ. The performance of both diagnostic tests is influenced by many factors, and a limitation of this review was incomplete reporting of characteristics that would potentially influence diagnostic performance such as operator experience (specified in just a third of included studies) and technical difficulties (three studies) [3, 28, 48].

iuMR is not without its limitations and our review demonstrated that iuMR overestimates the presence of abnormalities more frequently than failing to identify them. This could be explained by the nature of foetal iuMR in which the need for fast imaging compromises image quality. To the untrained eye artefacts from maternal breathing, foetal movement and image aliasing may potentially mimic or obscure pathology [52]. It is for this reason ‘experience’ should perhaps be defined by the number of foetal brain examinations reported.

The timing of USS in relation to iuMR imaging is also relevant in the assessment of both tests. The foetal brain develops rapidly and significant delay between the two examinations may influence the ability to diagnose accurately either because of natural brain development, increase in size of critical anatomical structures or because of disease progression. Thirteen out of 34 studies failed to report delay time, making an overall analysis of effect from this criteria unreliable.

The extent to which iuMR ultimately contributes to changes in management or in counselling regarding the pregnancy is also unclear as this was only reported in a small proportion of studies. Equally the impact of a wrong diagnosis made by iuMR was not defined in any study despite it occurring in 14/34 studies [18, 19, 21, 23, 26, 29, 33, 35, 36, 39–42, 44, 47].

Our review builds on the systematic reviews undertaken by Rossi et al. and van Doorn. Rossi identified 2323 potential studies published between years 2000 and the end of 2012 and reviewed 13 studies (710 foetuses), having excluded 2293 by title and abstract. Van Doorn searched for publications between years 1990 and March 2014 and identified 2748 and excluded 2577 by title and abstract with 27 studies (1184 abnormalities detected by USS but only 454 with ORD) reviewed. The differences of search dates and of exclusion criteria, described earlier, appear to be the factors resulting in the variation of studies reviewed by each study.

An important difference between the two is that Rossi restricted studies to those where outcomes were confirmed by a reference diagnosis, although chose to accept clinical examination as an ORD whereas van Doorns’ selection criteria did not require an ORD. A strength of our review was the requirement of an ORD for any outcomes included in the meta-analysis. As previously stated we excluded clinical examination as an ORD. Although this significantly reduced the number of outcomes available, we felt this was justified as most structural brain abnormalities, and consequently diagnostic accuracy, cannot be determined with certainty on clinical examination alone.

Our analysis included 34 studies, of which 15 were additional to those included in the previous reviews owing to more recent searches and differences in selection criteria such as unlimited year of publication or sample size within studies. Although Van Doorns’ searches were unrestricted by non-English publications or the requirement of an ORD, our review included more studies. This may be due to the limitation of sample size of less than 20 by van Doorn, resulting in six additional studies in this review, and the requirement of ‘adequate description of diagnoses’ which was not clearly defined by van Doorn.

Even with subtle differences in methods between all the reviews, findings were similar. Rossi reported that iuMR was accurately able to identify brain abnormalities in 94.3 % of included foetuses, van Doorn reported 80 % and our study 91 %, an increase of 15–20 % when compared to USS alone. Both Rossi and van Doorn reported that the highest proportion of disagreement between USS and iuMR was related to midline abnormalities, particularly the posterior fossa. iuMR was better able to diagnose abnormalities in this anatomical region, also consistent with the findings of this systematic review which incorporates a further four studies published since 2012.

Although heterogeneity was not quantified by Rossi and van Doorn, both reviews highlighted the inadequate reporting of study characteristics which may compromise the findings of all systematic reviews. In order to adequately assess the accuracy of a diagnostic test and determine its true benefit in clinical practice, optimal study design is necessary [51].

We believe replication of the previous reviews is both justified and necessary—it reassures that the minor differences in inclusion and exclusion criteria both at study selection and data extraction do not change the outcomes significantly, thus adding weight to the current evidence base. In spite of the different nature of all the studies, the diagnostic accuracy of iuMR was clearly superior across the studies but the heterogeneity identified may compromise these findings. The moderate level of heterogeneity identified by our review warranted further investigation but was prevented by insufficient reporting of study characteristics. Despite its increasing use in clinical practice, poor study design has previously brought into question the diagnostic capabilities of iuMR above that which is achieved by USS and its benefit in terms of guiding the management of pregnancy and further studies are needed [53]. For this reason we instigated the MERIDIAN [54] project, a large prospective study to investigate iuMR imaging in the diagnosis of foetal brain abnormalities to provide definitive evidence to guide future practice.

## Conclusion

When foetal brain abnormalities are suspected on USS, iuMR imaging is able to contribute significantly to the diagnostic pathway by both clarifying findings and increasing significantly the detection rate of abnormalities, particularly in midline and posterior fossa anomalies. Limitations of previous studies suggests that further investigation is still required to clarify the full impact of iuMR.

## Appendix 1

**Table 4:** Medline search strategy

	Database: Ovid MEDLINE(R) in-process & other non-indexed citations, Ovid MEDLINE(R) Daily, Ovid MEDLINE(R) and Ovid OLDMEDLINE(R) <1946 to Present> Search Strategy:-	Results (*n*)
1	Brain/ (381902)	381902
2	Abnormalities, Multiple/	36708
3	1 and 2	1518
4	(brain adj5 abnormalit$).mp.	6920
5	Ventriculomegaly.mp.	1506
6	"Agenesis of Corpus Callosum"/	1882
7	corpus callosum.mp.	15290
8	agenesis.mp.	10526
9	7 and 8	2720
10	Arnold-Chiari Malformation/	2609
11	Chiari malformation.mp.	3070
12	Dandy-Walker syndrome/	895
13	dandy walker.mp.	1258
14	3 or 4 or 5 or 6 or 9 or 10 or 11 or 12 or 13	16031
15	Fetus/ or Pregnancy/	720818
16	Prenatal Diagnosis/	31148
17	f?etus.mp.	115409
18	f?etal.mp.	306437
19	pregnan$.mp.	787645
20	in utero.mp.	21043
21	or/15-20	927013
22	Ultrasonography, Prenatal/ or Ultrasonography/	86794
23	ultraso$.mp.	313296
24	22 or 23	313296
25	Magnetic Resonance Imaging/	279781
26	(magnetic resonance imag$ or MRI).mp.	368017
27	25 or 26	368017
28	14 and 21 and 24 and 27	338
29	Comment/	584249
30	Letter/	840238
31	Editorial/	355577
32	(comment or letter or editorial).pt.	1331667
33	case reports.pt.	1686033
34	29 or 30 or 31 or 32 or 33	2843406
35	28 not 34	204

## Appendix 2

**Table 5:** Data items recorded for each study

Author, year
Title/objective
Publication type
Country of study
Language of publication
Study characteristics
Target population
Method of selection
Retrospective (R), prospective (P)
Total number in study
Number excluded + reasons
Final number included in review
Ultrasound details
Age of foetus at USS (weeks)
Level of USS: routine (R) or specialist (S)
Was USS equipment and technique described? (2D, 3D, TV etc.)
Experience of sonographer
Problems encountered? If yes specify
iuMR details
Age of foetus at iuMR
Time delay between USS and iuMR
Was iuMR technique and equipment described? (Magnet strength, use of sedation, sequences used etc.)
Fetal iuMR experience of reporting radiologist
Was the radiologist blinded to USS report?
Problems encountered? If yes specify
Outcome details
Reference standard used
Within 6 months?
Analysis: numbers confirmed by outcome reference diagnosis
iuMR and USS agreed and correct
iuMR and USS agreed but both wrong/missed significant finding
iuMR changed diagnosis (USS wrong)
USS additional information to iuMR (USS more exact)
USS changed diagnosis (iuMR wrong)
Number where iuMR changed counselling
Number where iuMR changed management
Specific abnormalities where iuMR was most accurate/useful
Specific abnormalities where iuMR least accurate

## Abbreviations

iuMR: in utero magnetic resonance
ORD: outcome reference diagnosis
PRISMA: Preferred Reporting Items for Systematic Reviews and Meta-Analyses
PROSPERO: International Prospective Register of Systematic Reviews
USS: ultrasound scanning
